# 653. BERILS (Burn Ergonomic Intraoperative Load Score) – A Proposed Scoring System to Predict Ergonomic Stress

**DOI:** 10.1093/jbcr/irag033.490

**Published:** 2026-04-06

**Authors:** Sheiryl Gica, Philip H Chang

**Affiliations:** New York Presbyterian Hospital Weill Cornell, Rego Park, NY; William Randolph Hearst Burn Center, NY, New York

## Abstract

**Introduction:**

The surgical care of burn patients involves laborious efforts by a highly specialized operative team, operating frequently under ergonomically challenging conditions. Anecdotally, non-burn OR personnel express reluctance to assist with burn surgical cases because of the perceived physical and emotional stresses. Our team has embarked on a multi-stage research project to specify and quantitate the various factors contributing to ergonomic stress of the burn operative team with an eventual goal of designing team interventions to decrease this workload. This study aimed to create a novel scoring system to predict the ergonomic stress of the burn operative team.

**Methods:**

This is a quality improvement project to improve ergonomics in burn surgery. The ergonomic behavior of the burn surgical team at a single high volume ABA-verified burn center was monitored and assessed using a direct observation method over a 6 month period. Based on the observations, a multivariate scoring system was formulated.

**Results:**

It was determined by direct observation method that burn surgeries vary with regards to the ergonomic workload, level of ergonomic risk. A total of 283 burn surgeries were performed in 6 months between 2 burn surgeons. 159 patients weighed >50 lbs. Average of continuous 2-hour long of prolonged standing were observed. Variables identified as contributing to ergonomic stress included peak room temperature, predicted duration of case, number of extremities to be operated on, % TBSA to be excised, weight of patient, number of patient positions needed for case, harvesting for autograft, circumferential burn injury on extremities, and need for uncommon or rare multi-step procedures.

**Conclusions:**

Disregarding work-related musculoskeletal discomfort, burn surgical teams were observed to perform ergonomically challenging feats to provide the highest surgical care. Knowledge of direct observation did not alter operative team behavior. Following this ergonomic assessment/monitoring and using this BERIL score, a prospective study of the ergonomic stresses endured of the burn operative team will be the next step. Future directions will involve interventions to decrease the ergonomic stress, thus increasing the longevity of the burn operative teams and decreasing turnover of personnel from workplace injuries.

**Applicability of Research to Practice:**

Applicable to Practice.

**Funding for the study:**

N/A.

